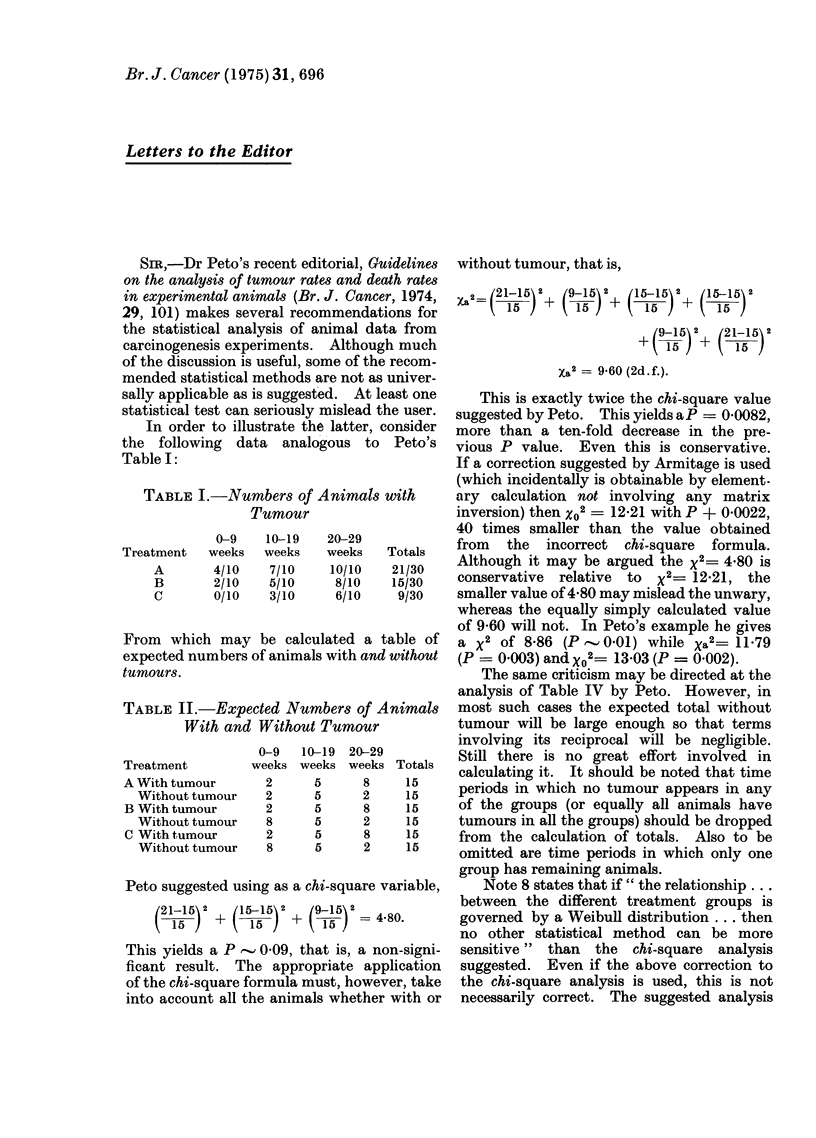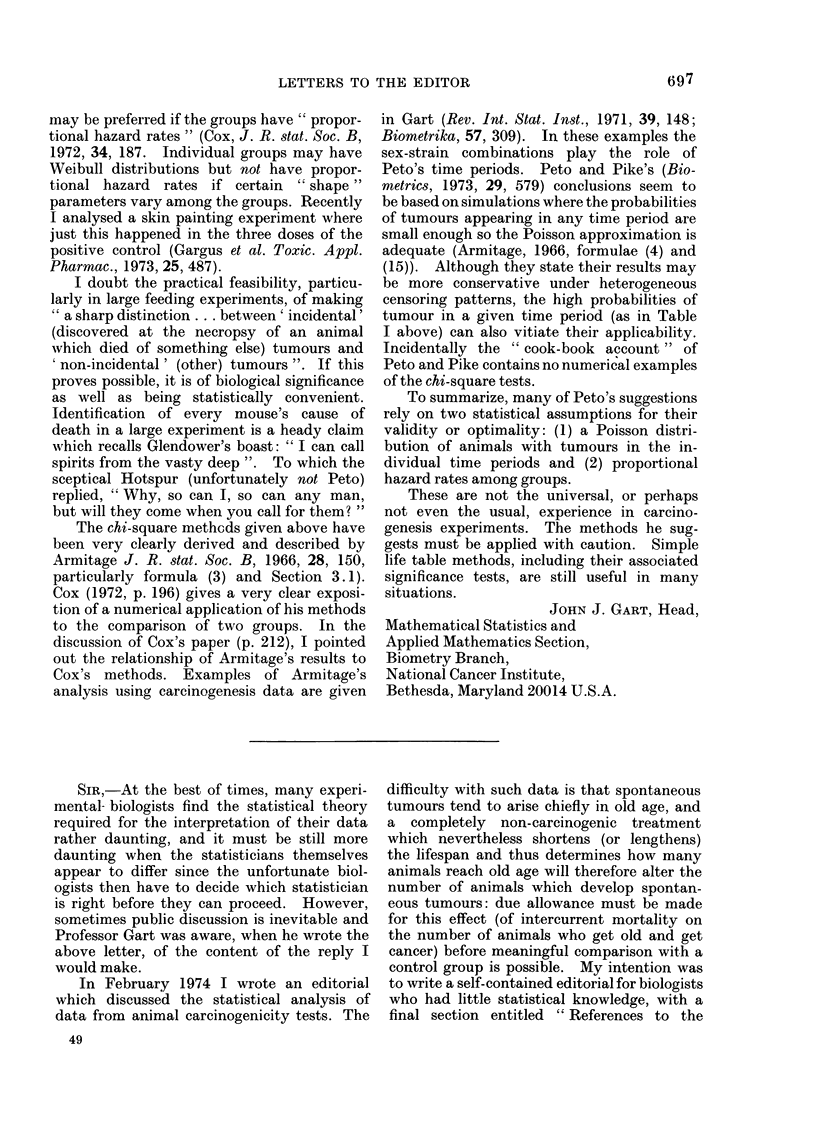# Letter: Analysis of tumour rates and death rates in experimental animals.

**DOI:** 10.1038/bjc.1975.119

**Published:** 1975-06

**Authors:** J. J. Gart


*Br. J. Cancer* (1975) **31**, 696

### Letters to the Editor

SIR,—Dr Peto's recent editorial, *Guidelines on the analysis of tumour rates and death rates in experimental animals* (*Br. J. Cancer*, 1974, **29**, 101) makes several recommendations for the statistical analysis of animal data from carcinogenesis experiments. Although much of the discussion is useful, some of the recommended statistical methods are not as universally applicable as is suggested. At least one statistical test can seriously mislead the user.

In order to illustrate the latter, consider the following data analogous to Peto's Table I:

TABLE I.—*Numbers of Animals with Tumour*

| Treatment | 0–9 weeks | 10–19 weeks | 20–29 weeks | Totals |
|---|---|---|---|---|
| A | 4/10 | 7/10 | 10/10 | 21/30 |
| B | 2/10 | 5/10 | 8/10 | 15/30 |
| C | 0/10 | 3/10 | 6/10 | 9/30 |

From which may be calculated a table of expected numbers of animals with *and without tumours*.

TABLE II.—*Expected Numbers of Animals With and Without Tumour*

| Treatment | 0–9 weeks | 10–19 weeks | 20–29 weeks | Totals |
|---|---|---|---|---|
| A With tumour | 2 | 5 | 8 | 15 |
| Without tumour | 2 | 5 | 2 | 15 |
| B With tumour | 2 | 5 | 8 | 15 |
| Without tumour | 8 | 5 | 2 | 15 |
| C With tumour | 2 | 5 | 8 | 15 |
| Without tumour | 8 | 5 | 2 | 15 |

Peto suggested using as a *chi*-square variable,

$$\left(\frac{21-15}{15}\right)^2 + \left(\frac{15-15}{15}\right)^2 + \left(\frac{9-15}{15}\right)^2 = 4\cdot80.$$

This yields a $P \sim 0\cdot09$, that is, a non-significant result. The appropriate application of the *chi*-square formula must, however, take into account all the animals whether with or without tumour, that is,

$$\chi_a^2 = \left(\frac{21-15}{15}\right)^2 + \left(\frac{9-15}{15}\right)^2 + \left(\frac{15-15}{15}\right)^2 + \left(\frac{15-15}{15}\right)^2 + \left(\frac{9-15}{15}\right)^2 + \left(\frac{21-15}{15}\right)^2$$

$$\chi_a^2 = 9\cdot60 \text{ (2d.f.)}.$$

This is exactly twice the *chi*-square value suggested by Peto. This yields a $P = 0\cdot0082$, more than a ten-fold decrease in the previous $P$ value. Even this is conservative. If a correction suggested by Armitage is used (which incidentally is obtainable by elementary calculation *not* involving any matrix inversion) then $\chi_0^2 = 12\cdot21$ with $P + 0\cdot0022$, 40 times smaller than the value obtained from the incorrect *chi*-square formula. Although it may be argued the $\chi^2 = 4\cdot80$ is conservative relative to $\chi^2 = 12\cdot21$, the smaller value of 4·80 may mislead the unwary, whereas the equally simply calculated value of 9·60 will not. In Peto's example he gives a $\chi^2$ of 8·86 ($P \sim 0\cdot01$) while $\chi_a^2 = 11\cdot79$ ($P = 0\cdot003$) and $\chi_0^2 = 13\cdot03$ ($P = 0\cdot002$).

The same criticism may be directed at the analysis of Table IV by Peto. However, in most such cases the expected total without tumour will be large enough so that terms involving its reciprocal will be negligible. Still there is no great effort involved in calculating it. It should be noted that time periods in which no tumour appears in any of the groups (or equally all animals have tumours in all the groups) should be dropped from the calculation of totals. Also to be omitted are time periods in which only one group has remaining animals.

Note 8 states that if " the relationship . . . between the different treatment groups is governed by a Weibull distribution . . . then no other statistical method can be more sensitive " than the *chi*-square analysis suggested. Even if the above correction to the *chi*-square analysis is used, this is not necessarily correct. The suggested analysis may be preferred if the groups have " proportional hazard rates " (Cox, *J. R. stat. Soc. B*, 1972, **34**, 187. Individual groups may have Weibull distributions but *not* have proportional hazard rates if certain " shape " parameters vary among the groups. Recently I analysed a skin painting experiment where just this happened in the three doses of the positive control (Gargus *et al. Toxic. Appl. Pharmac.*, 1973, **25**, 487).

I doubt the practical feasibility, particularly in large feeding experiments, of making " a sharp distinction . . . between ' incidental ' (discovered at the necropsy of an animal which died of something else) tumours and ' non-incidental ' (other) tumours ". If this proves possible, it is of biological significance as well as being statistically convenient. Identification of every mouse's cause of death in a large experiment is a heady claim which recalls Glendower's boast: " I can call spirits from the vasty deep ". To which the sceptical Hotspur (unfortunately *not* Peto) replied, " Why, so can I, so can any man, but will they come when you call for them? "

The *chi*-square methods given above have been very clearly derived and described by Armitage *J. R. stat. Soc. B*, 1966, **28**, 150, particularly formula (3) and Section 3.1). Cox (1972, p. 196) gives a very clear exposition of a numerical application of his methods to the comparison of two groups. In the discussion of Cox's paper (p. 212), I pointed out the relationship of Armitage's results to Cox's methods. Examples of Armitage's analysis using carcinogenesis data are given in Gart (*Rev. Int. Stat. Inst.*, 1971, **39**, 148; *Biometrika*, **57**, 309). In these examples the sex-strain combinations play the role of Peto's time periods. Peto and Pike's (*Biometrics*, 1973, **29**, 579) conclusions seem to be based on simulations where the probabilities of tumours appearing in any time period are small enough so the Poisson approximation is adequate (Armitage, 1966, formulae (4) and (15)). Although they state their results may be more conservative under heterogeneous censoring patterns, the high probabilities of tumour in a given time period (as in Table I above) can also vitiate their applicability. Incidentally the " cook-book account " of Peto and Pike contains no numerical examples of the *chi*-square tests.

To summarize, many of Peto's suggestions rely on two statistical assumptions for their validity or optimality: (1) a Poisson distribution of animals with tumours in the individual time periods and (2) proportional hazard rates among groups.

These are not the universal, or perhaps not even the usual, experience in carcinogenesis experiments. The methods he suggests must be applied with caution. Simple life table methods, including their associated significance tests, are still useful in many situations.

JOHN J. GART, Head,
Mathematical Statistics and
Applied Mathematics Section,
Biometry Branch,
National Cancer Institute,
Bethesda, Maryland 20014 U.S.A.